# Endoscopic treatment of a hepatic cyst, buried lumen-apposing metal stent, and liver abscess: the good, the bad, the ugly

**DOI:** 10.1016/j.vgie.2023.07.006

**Published:** 2023-08-24

**Authors:** Andrew Canakis, Shayan S. Irani

**Affiliations:** 1 Division of Gastroenterology and Hepatology, University of Maryland School of Medicine, Baltimore, Maryland; 2 Division of Gastroenterology and Hepatology, Virginia Mason Medical Center, Seattle, Washington

## Abstract

Video 1 Demonstration of the management of a recurrent symptomatic hepatic cyst that was complicated by a buried lumen-apposing metal stent in the gastric antrum and a liver abscess.

Demonstration of the management of a recurrent symptomatic hepatic cyst that was complicated by a buried lumen-apposing metal stent in the gastric antrum and a liver abscess.

## Introduction

Hepatic cysts represent a heterogenous group of lesions that typically have a benign course. However, a minority of these cysts can cause symptoms related to abdominal pain, nausea, vomiting, or bloating. A larger cyst can lead to adverse events related to infection, spontaneous hemorrhage, compression of the biliary tree, or potential malignant transformation.^1^^,^^2^ These symptomatic cysts are ideally managed with percutaneous aspiration or surgery. In this video, we describe a patient who underwent multiple surgical attempts over a 33-year period to completely eradicate a recurrent hepatic cyst that was ultimately managed endoscopically despite adverse events by a buried lumen-apposing metal stent (LAMS) and a liver abscess (Video 1, available online at www.videogie.org).

## Case

A 65-year-old woman was referred for her fourth painful recurrence of a hepatic cyst. The 25-cm cyst was initially diagnosed in 1983, when she underwent a partial cyst resection and marsupialization. She remained asymptomatic until 2007, when she developed a painfully recurrent cyst measuring 8.5 cm. Surgical resection was unsuccessful and the cyst contents were bilious and mucoid, suggesting a possible cystadenoma of the wall, for which the lesion was ablated with alcohol. In 2010, surgeons were unable to mobilize a Roux limb to the liver; consequently, partial resection and cystogastrostomy were done to relieve the pain for up to 3 years. However, she developed recurrence of the cyst in 2014. Her fourth surgery attempt included a partial hepatectomy, cyst resection, marsupialization, and alcohol ablation, but these failed. She was then referred for endoscopic therapy.

An initial attempt at EUS-guided cystogastrostomy in 2014 (with a non-cautery enhanced LAMS) resulted in severe bleeding after balloon dilation, with the cyst falling away from the gastric wall resulting in hemorrhagic shock and cardiac arrest from a bleeding left hepatic artery that was embolized. After a year of ongoing pain and resolution of a retroperitoneal hematoma, she requested another attempt at endoscopic drainage.

**Figure 1:**
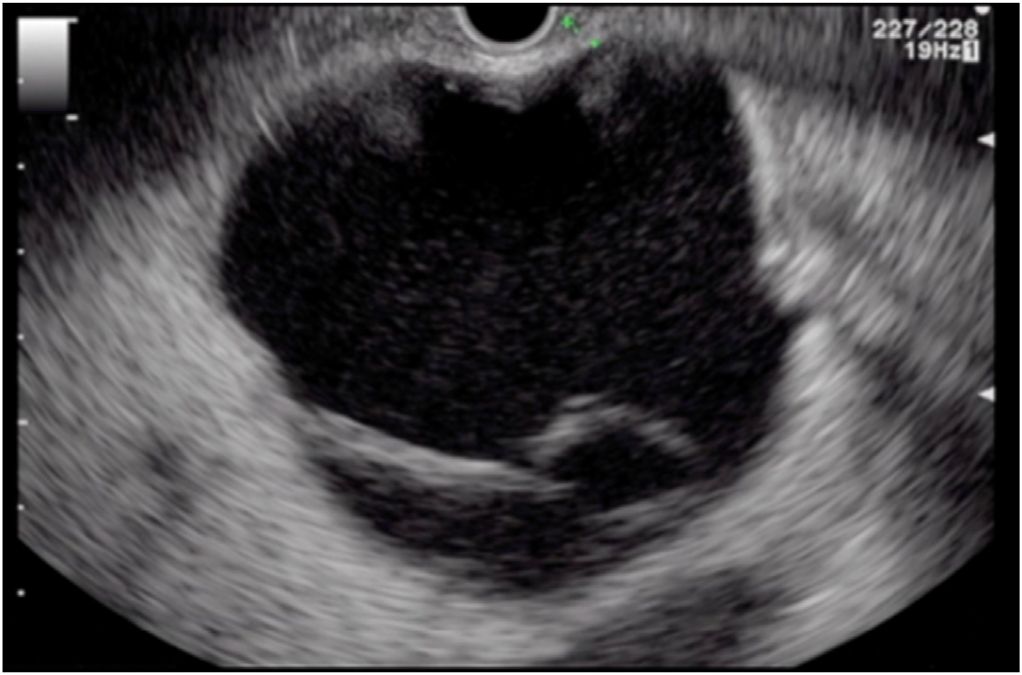
EUS demonstrating a septated hepatic cyst abutting the gastric wall by less than 1 cm.

**Figure 2:**
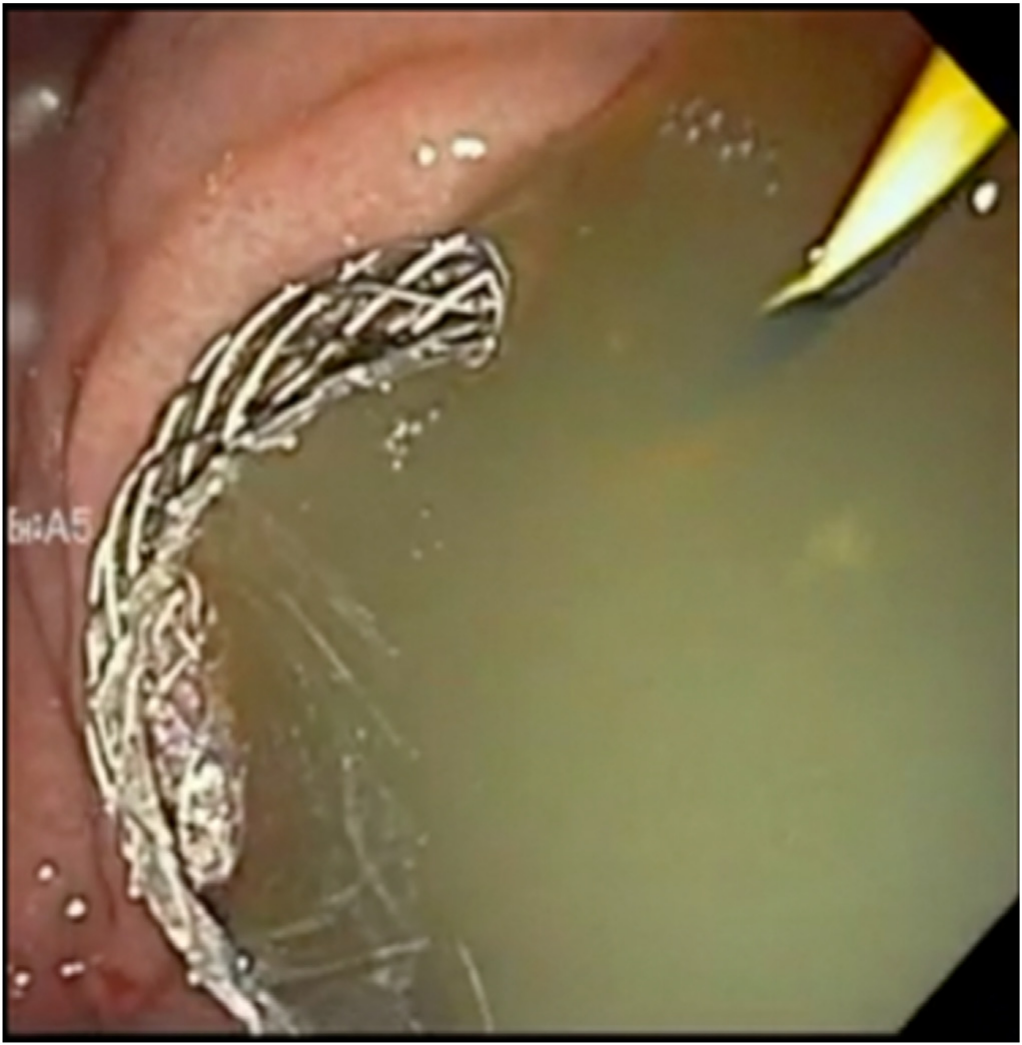
Cyst fluid draining after lumen-apposing metal stent deployment.

On repeat EUS, the wall distance between the cyst and the gastric wall was less than 1 cm, and a 19-gauge needle was used to puncture the cyst again—this time closer to the antrum, where the surgical cystogastrostomy had been performed previously (Fig. 1). A 0.035-inch guidewire was again coiled in the cyst. However, we were unable to dilate the tract with a balloon or catheter (because of the thick scar), and a cystotome was used to gain access followed by successful balloon dilation of the tract. Afterward, successful deployment with a 15- × 10-mm LAMS (Boston Scientific, Natick, Mass, USA) was performed (Fig. 2). A 7F × 3-cm double-pigtail stent was then deployed through the LAMS into the cyst to reduce the risk of granulation overgrowth into the stent.

**Figure 3:**
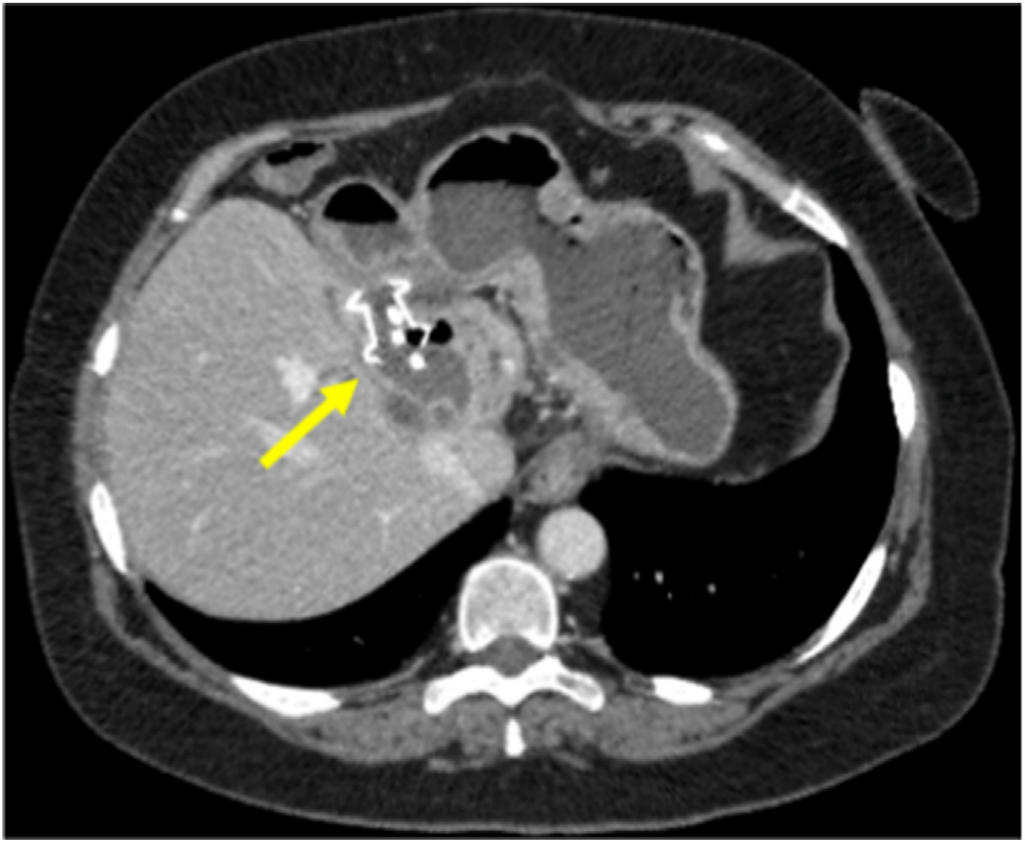
A CT scan showing an undrained portion of the cyst concerning for an abscess.

**Figure 4:**
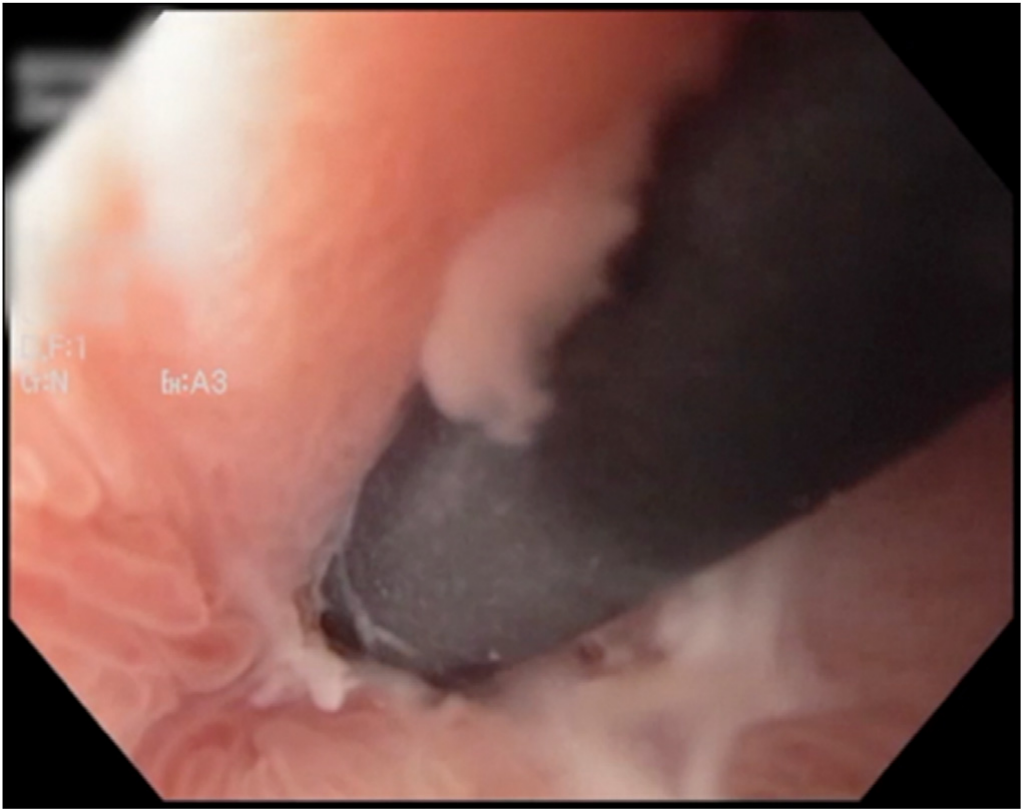
Subsequent endoscopy demonstrating a completely buried lumen-apposing metal stent in the antrum with only the pigtail stent visible.

**Figure 5:**
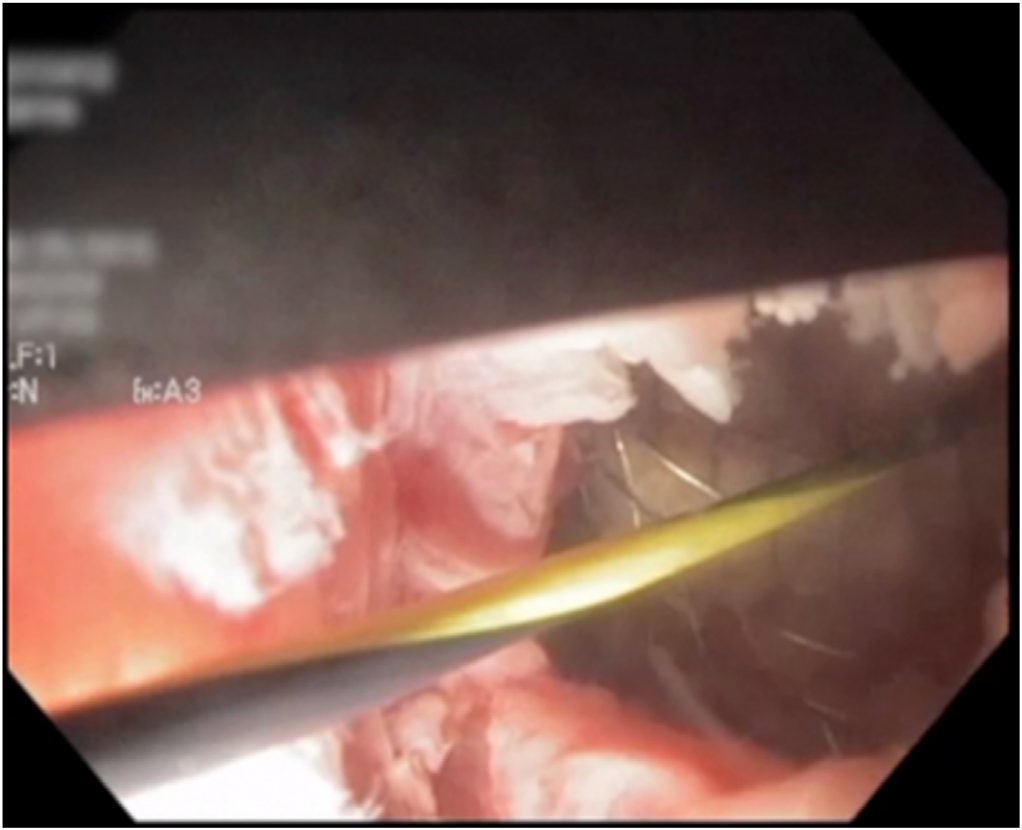
Buried lumen-apposing metal stent visible after balloon dilation.

**Figure 6:**
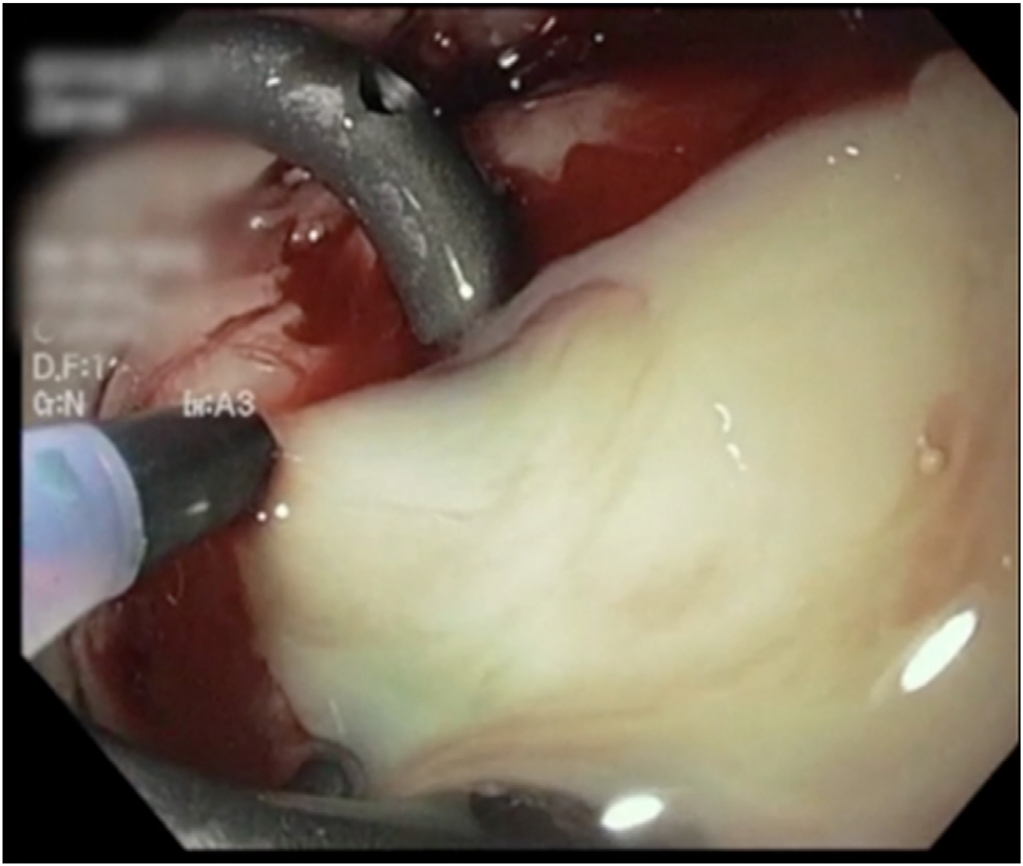
Purulent drainage of the undrained portion of the cyst following injection with a sclerotherapy needle.

After being asymptomatic for 4 weeks, she developed a low-grade fever and pain. A CT scan showed an undrained portion of the cyst concerning for an abscess (Fig. 3). A subsequent upper endoscopy demonstrated a buried LAMS and only the 7F pigtail stent being visible (Figs. 4 and 5). An ERCP extraction balloon with a guidewire was used to access the liver cyst, where purulent discharge confirmed findings of an abscess. Dilation with a 12- to 15-mm dilating balloon (CRE Balloon; Boston Scientific) allowed visualization of the buried LAMS, and the endoscope was then advanced over the guidewire into the hepatic cyst/abscess. Because of concern for an additional undrained component of the cyst (as seen on the CT scan), a 23-gauge sclerotherapy needle was used to puncture the wall of liver facing the endoscope. Contrast injection demonstrated the residual, undrained portion of the cyst, and on needle withdrawal, pus emerged (Fig. 6). The pigtail stent and LAMS were removed. This residual undrained component of the cyst was then drained. After balloon dilation, the cyst wall was biopsied, followed by the placement of three 7F × 5-cm double-pigtail stents into the deepest portion of the cyst. Biopsies of the cyst wall demonstrated benign liver tissue with no evidence of adenoma. A CT scan 3 years postprocedure showed the stents still in situ. Since the procedure, she has remained asymptomatic over a 7-year follow-up period with no recurrence of abdominal pain or infections attributable to the cyst.

## Conclusion

Ideally located, symptomatic hepatic cysts in nonsurgical patients can be treated with EUS-guided cystogastrostomy using a LAMS. One must drain all components of the cyst and be aware of the risk of buried LAMS in the antrum. Management of patients with a large symptomatic hepatic cyst should be carried out in a multidisciplinary setting. This case demonstrated that long-term relief of symptomatic cysts can be achieved with a cystogastrostomy. The procedure and indication require further study before being widely adopted, and the risk of life-threatening bleeding (as with any transhepatic puncture) should be discussed with the patient.

## Disclosure

Dr Irani is a consultant for Boston Scientific, CONMED, and Gore. Dr Canakis disclosed no financial relationships.

## Supplementary data

Video 1 Demonstration of the management of a recurrent symptomatic hepatic cyst that was complicated by a buried lumen-apposing metal stent in the gastric antrum and a liver abscess.
